# Political cycles: Beyond rational expectations

**DOI:** 10.1371/journal.pone.0203390

**Published:** 2018-10-11

**Authors:** Frank Bohn

**Affiliations:** Nijmegen Centre for Economics (NiCE), Institute for Management Research (IMR), Radboud University, Nijmegen, The Netherlands; Universidad de Castilla-La Mancha, SPAIN

## Abstract

**Motivation and method:**

Existing rational expectations models cannot satisfactorily explain why political budget manipulations systematically raise re-election chances and only occur in “specific contexts”. This paper offers a theoretical explanation by including unsophisticated voters into an opportunistic political cycle model; unsophisticated voters are unable to take the optimal behaviour of other agents (fully) into account, but may, nonetheless, vaguely suspect government deception.

**Results:**

First, rationally expected manipulations are, on average, fruitless in equilibrium. By including unsophisticated voters we can, however, corroborate empirically found electoral effects of political budget manipulations. Second, unsophisticated voters become anxious and suspicious in an intransparent or uncertain world, but the government tries to “outperform” their scepticism by increasing budget manipulations in order to appear more competent and, ultimately, increase re-election chances. It is, therefore, not surprising that political budget cycles are observed in countries suffering from intransparencies such as developing countries or new democracies. Third and in addition, the model presented here predicts that political opportunism produces, unintentionally, a countercyclical policy effect in election years, thereby, for instance, alleviating the typical problem of policy procyclicality in developing countries.

**Additional contribution:**

The paper also offers a theoretical explanation for political distortions found in forecasts by US states. Based on overly optimistic revenue forecasts the incumbent state government can conduct expansionary fiscal policies in order to appear more competent prior to an upcoming election. Since the resulting deficit can only be observed afterwards, the government can effectively circumvent a constitutional balanced budget constraint. As a result, there are political forecast and budget cycles in the state. More generally, however, these findings may also apply to European countries where balanced budget constraints are or will be in place (for instance the debt brakes in Switzerland and Germany); similarly, they apply to the supra-national European Fiscal Compact of the European Union.

## Introduction

It has become conventional wisdom that small deviations from rationality can make significant differences to economic equilibria as suggested by Akerlof and Yellen [1]. If a share of the population fails to respond optimally to changes in the environment, outcomes may be significantly different to those under full rationality by all agents. Akerlof and Yellen [1] define behaviour as near-rational, if a shock leads to first-order welfare effects for society, but only second-order losses for individuals who may then exhibit “inertial behavior”, i.e. not respond fully rationally to the shock. They derive the rationale for near-rationality from the Envelope Theorem and present various examples from macroeconomics and industrial organisation. Near-rationality in monetary policy is discussed, for instance, by Woodford [2] and Adam and Woodford [3]. The (“distorting”) effect of non-maximising behaviour on the equilibrium has been studied in numerous papers; some of the earlier ones are Haltiwanger and Waldman [4], Russell and Thaler [5] [6], Berry [7] and Fudenberg and Maskin [8]. Going beyond near-rationality in his Nobel speech, Akerlof [9] presents a plethora of *behavioural reasons* for explaining “six [empirically observed] macroeconomic phenomena” that New Classical economics and its rational expectations paradigm ([10, 11]) cannot account for. Those *behavioural reasons* are, for instance, asymmetric information, rationing due to efficiency wages, the use of rules of thumb involving money illusion, downward wage rigidity as implied by prospect theory, a lack in self-control as embodied in hyperbolic discounting, or the role of identity in explaining self-destructive behaviour.

Another *behavioural reason* for deviations from rational expectations outcomes is encountered in the voting literature. Unlike sophisticated voters [12], unsophisticated voters do not take the rational behaviour of the rest of society into account. Unsophisticated voters take decisions on the basis of a distorted perception of reality or do not link their perception to their voting decision, whereas sophisticated voters are empirically found to (typically) form correct expectations and vote rationally according to Alt, Lassen and Marshall [13]. Aidt (p. 359) [14], concludes that “voters may not be fully informed but they know enough to make rational and informed decisions”, whereas Aidt and Dutta (p. 354) [15] contend that “other groups (the poor and uneducated) … are … unlikely to cast rational … votes”. In Bohn [16], voters can be mislead by the government because it can use its incumbency advantage to manipulate voters’ perceptions of the deficit. In Maloney and Pickering [17], sophisticated voters can distinguish (long-term) trend growth from short-term economic cycles, whereas unsophisticated voters only respond to raw GDP data.

This paper introduces unsophisticated voters into the political cycle literature because they can explain empirical phenomena that cannot be explained satisfactorily by existing rational expectations models. First, there is empirical evidence that political budget manipulations *systematically* raise re-election chances. But rational expectations models cannot produce this result by construction; if manipulations are rationally anticipated, then they will, at least on average, be fruitless in equilibrium. Second, there is no (rational expectations) explanation why political budget cycles should be “context-conditional”. However, empirical evidence shows that political budget manipulations only occur in “specific contexts”, for instance in countries with fiscal or government intransparencies, in developing countries, or in new democracies (see next section). The institutional settings that are conducive to political budget cycles have one element in common though; they give rise to perceived uncertainties about the functioning of institutions. Unsophisticated voters are unable to take the optimal behaviour of others (fully) into account, but become more anxious when the world around them turns more intransparent or uncertain. As a result, they are likely to become more suspicious about being deceived by the government. The government, in turn, will increase its political budget manipulation in order to appear more competent and, ultimately, be able to increase its re-election chances.

In addition to providing the theoretical rationale for the aforementioned empirical phenomena, this paper also finds a new theoretical result which has yet to be verified empirically. The model presented here predicts that political opportunism produces, unintentionally, a countercyclical policy effect. It is clear that a government will adjust its fiscal manipulation, if its (honest) expectation of economic growth changes. But this paper suggests that the fiscal manipulation does not change 1-for-1 with the expected change in economic growth. Take a recession. If the government cut spending too much, it could lose the support of unsophisticated voters (who may be unaware of a looming recession and/or unable to fully disentangle a recession from government policies). The government is, therefore, willing to accept a higher deficit, although it is costly. This is a countercyclical effect compared to the fiscal manipulation when there is no recession. In case of a boom, the situation reverses. The government has additional resources that can be used for appearing more competent. Given that re-election chances are increased when spending goes up, it is optimal for the government to reduce its expected (costly) deficit somewhat relative to the deficit it would have desired without boom expectations. Again, this is a countercyclical effect. Note that this is not countercyclical Keynesian policy, but an additional effect motivated by opportunistic government behaviour in election years. It is a positive analysis of (additional) conditions conducive to countercyclical policies. As for fiscal consolidations, it augments the discussion of conditions and problems of such consolidations as presented, for instance, by Perotti [18].

This result goes beyond the mere existence of political budget cycles (for the standard case of no recession expectations) as confirmed by ample empirical evidence. It says that expected recessions *increase* such a political budget cycle. This is a new theoretical finding, which can, in principle, be tested empirically. More generally, it is claimed that opportunistic government behaviour has a stabilising effect in both booms and recessions that occur during an election year. It is particularly good news for many developing countries that used to suffer and mostly still suffer from procyclical fiscal policies (for instance [19]); at least during a recession occurring in an election year, the economic contraction would be somewhat alleviated. The mechanism is similar to the one in Aidt and Dutta [15] [20]. There, “economic [regulation] policy is more inefficient during booms than during recessions” ([15], p. 354). Policy is, therefore, employed countercyclically. Here, it is the fiscal policy that is more costly and more inefficient in booms rather than recessions (in terms of affecting the government’s re-election chances). As a result, opportunistic government behaviour produces a countercyclical policy effect, though not with that intention in mind.

This paper also explains forecast manipulations observed in US states. Unsophisticated voters can be impressed by budget manipulations which are made possible by forecast manipulations; with overly optimistic growth and, thereby, exaggerated revenue forecasts the government can expand its spending despite a constitutional balanced budget constraint. Finally, the paper contributes to the policy discussion of European (supra-) national fiscal rules.

The remainder of the paper is structured as follows. The methods section discusses recent developments in the literature and the motivation for including unsophisticated voters in an electoral forecast cycle model. The model section comprises an outline of the model and its general solution. The differences to previous political budget cycle models are pointed out; it is shown that the winning probability can be affected by political manipulations. The results section discusses the role of unsophisticated voters in explaining “context-conditional” political cycles and the government’s countercyclical policy response to changing recession expectations as well as the theoretical and empirical implications of this new result.

## Methods

Politicians have long been suspected of manipulating electoral outcomes to their own advantage by creating short-term economic boosts and/or by improving citizens’ economic conditions just before an election. Yet, the evidence for such opportunistic politico-economic cycles is mixed. First, monetary policy-induced cycles as originally suggested by Nordhaus [21] are clearly rejected empirically [22]. Second, there is plenty of evidence on electoral manipulations in fiscal policies. Political cycles can be found, for instance, in debt, public expenditures, especially transfers, and expenditure shares (for instance [23, 24]). Third, more recently, several studies have argued that opportunistic political budget cycles (PBCs) are “context-conditional”. The term was coined by Francese [25]. A literature survey is provided by de Haan and Klomp [26]. For the context of Israeli local elections, Brender [27] finds the opposite effect; refraining from short-termism may actually increase a politician’s re-election chances. Responsible fiscal policies had a significant and positive effect in the 1998 local elections, though not in two prior elections. Nonetheless, there is overwhelming evidence on “context-conditional” political budget cycles. Many authors claim that political budget cycles are particularly relevant in developing countries ([28] [29] [30] [31] [32]). However, Brender and Drazen [33] reexamine the sample used in [31] and find that the evidence points to new democracies rather than developing countries. Others find that political budget cycles depend on, for instance, low levels of fiscal or government transparency [34] [35]. Chang [36] and Streb, Lema and Torrens [37] find that they are also affected by the political system; Alt and Mooney [38] link them to the electoral system. Veiga, Veiga and Morozumi [39] confront several of these conditioning factors and find that media freedom is key in reducing the incidence of PBCs.

### Suspicious unsophisticated voters

This paper presents a framework which extends the previous literature in two directions as shown in Table 1 below. The first extension refers to the types of voters that are included in the model; read the table from left to right. This paper introduces the concept of *unsophisticated* voters into the theoretical political cycle literature. Unsophisticated voters are not (fully) rational. Nonetheless, they might have a vague idea about what they should expect for the future. They may be sensitive to the institutional “context” they encounter. If they perceive the functioning of the government and the world around them as less transparent and more uncertain, they may be more suspicious of government actions. The link between each institutional “context” and the degree of uncertainty and suspicion created is likely to vary in each “context” and is beyond the scope of this paper. What this paper can show is that any “context-specific” suspicions held by unsophisticated voters may prompt the government to respond optimally to its expectation of such suspicions by employing its instrument(s) of manipulation. Unsophisticated voters’ suspicions cannot be captured in a model where voters are assumed to form rational expectations of future government behaviour or ability as, for instance, in Lohmann [40] and Shi and Svensson [31]. Instead, this paper argues that suspicions held by unsophisticated voters (caused by institutional “context-specific” intransparency and uncertainty) are relevant and can explain government manipulations. If we follow this line of reasoning, it is not surprising that political cycles are more likely to occur, for instance, in new democracies and developing countries (as well as other “contexts”), since intransparencies and uncertainties are often very pronounced there.

**Table 1:** Categories of political cycle models and types of voters.

Types of Voters: Political … Cycles	Only Sophisticated Voters:		Informed and Uninformed Sophisticated as well as Unsophisticated Voters:
	Only Uninformed Voters:	Informed and Uninformed Voters:	
… Business …	Lohmann, 1998 Rogoff and Sibert, 1988		
… Budget …	Rogoff, 1990	Shi and Svensson, 2006	
… Forecast …			This paper

Unsophisticated voters do not or cannot make good or full use of the available information; they may misperceive information or ignore it for lack of interest; or they may misunderstand the link between economic conditions and the available information (see also first part of results section). As a consequence, they take decisions on the basis of a distorted perception of reality. It should be noted that such deviations from rational expectations were already considered in the first (seminal) article on rational expectations by Muth [10]. Unsophisticated voters may (or may not) be able to respond to the current government policy, but certainly not in a (fully) rational way. In all other respect, they are not different from sophisticated voters. Like any ordinary voter they are interested in voting for the candidate who they expect to deliver the highest utility for them. In other words, all voters, sophisticated or not, are prospective. In the literature, prospective voting has sometimes been associated indistinguishably (and confusingly) with rational voting because voters were modelled, at the same time, to be able to anticipate developments or changes in candidates’ behaviour or deviations from pre-announced policy platforms after the elections. Here, rational expectations of sophisticated voters refer to government manipulations that occur *before* elections; and there is nothing that sophisticated voters could anticipate about the future (*after* elections) that unsophisticated voters would not know about either.

An unsophisticated voter is different to a voter in the adaptive-expectations framework suggested by Nordhaus [21], where naive voters base their electoral choice on the past experience with the behaviour of their government. An unsophisticated voter is also very different to the *uninformed* voter suggested by Lohmann [40] and Shi and Svensson [31]—see Table 1. The political business cycle model by Rogoff and Sibert [41] and the political budget cycle model by Rogoff [42] can also be entered into the “only uninformed voters” column. To the best of my knowledge, there is, however, no paper that could be added to the last column or to the bottom row. – [41] and [42] are signalling models (unlike [40] and [31]) in which the government can exploit an information advantage; the government has complete knowledge over its own competence at the moment it takes decisions. As a consequence, we obtain the surprising result (at least when contrasted with the real world) that governments which are incompetent will not (be able to) signal, i.e. not be able to manipulate—a typical result in signalling models though.

In this paper we include informed and uninformed voter suggested by Shi and Svensson [31] (and Lohmann [40]). Their informed voters are rational in that they can deduce government ability. However, their uninformed voters are also rational in that they are able to form rational expectations about government behaviour. Both, informed and uninformed voters, are actually very sophisticated. By including these sophisticated types in addition to unsophisticated voters differences to these earlier papers can be clearly identified. With the inclusion of unsophisticated voters it is now possible to explain the empirical finding (to my knowledge for the first time in a theoretical model) that the incumbent’s vote share increases *systematically* with electoral manipulations conducted by the government—as found by, for instance, Aidt, Veiga and Veiga [43], Akhmedov and Zhuravskaya [35], and de Haan and Klomp [44]. Furthermore, increased suspicions by unsophisticated voters amplify the political cycle; government manipulations must be more potent than the suspicions held by unsophisticated voters in order to be effective. If more political or economic intransparencies do really make voters more suspicious, then this result can explain the prominence of political manipulation in countries suffering from intransparencies including developing countries and new democracies.

### Political forecast cycles

The second extension refers to the type of political manipulation (read aforementioned Table 1 downwards). This paper introduces forecast manipulations as a government tool into the theoretical political cycle literature. The model captures the specific circumstances of US states, most of which have to adhere to constitutional balanced budget constraints. The resulting political forecast cycle model provides a theoretical explanation for empirical findings by Boylan [45]. The specific forecast manipulations observed in US states are outlined below. More generally, the results relate to European Union local and state governments as well as the European discussion of (supra-) national fiscal rules—also discussed below.

The paper argues that a constitutional balanced budget constraint does not prevent deficit making, albeit, possibly, avoid the build-up of long-term debt. In election years, the incumbent claims to fulfill the balanced budget requirement *ex ante*, albeit based on overly optimistic growth and, thereby, exaggerated revenue forecasts. *Ex post*, it turns out—to the government’s mock surprise—that the budget cannot be balanced. But the government could expand spending despite the constitutional balanced budget constraint, thereby increasing its re-election chances. The government machinations can only be effective because the incumbent can (partially) hide the forecast manipulations from part of the electorate. Here is the hidden effort which constitutes the moral hazard problem. As debt is costly, it will be repaid in off-election years, thereby not only producing a political forecast cycle, but also a budget cycle. The model here exhibits a mechanism similar to the one in other moral hazard political cycle models. In Shi and Svensson [31] or Lohmann [40], for instance, politicians’ manipulations are aimed at expanding the budget or the money supply, respectively, in order to increase re-election chances. There, the incumbent can, however, not raise her re-election probability above 50% due to the rational expectations assumption for uninformed voters—as already mentioned earlier. Despite the forecast cycle perspective we can replicate their result by setting the share of unsophisticated voters to zero; their papers are special cases of this paper.

The effect produced by suspicious unsophisticated voters on political cycles is studied against the backdrop of constitutional balanced budget constraints and forecast manipulations, but its implications are more general. The sovereign debt and banking crisis in the Eurozone revealed successful government attempts to manipulate the international public’s perception of a country’s fiscal situation. Greece and other European countries cheated to hide “excessive” public debt and/or deficits so that they could gain access to the European Monetary Union and/or fulfill the criteria laid out by the European Stability and Growth Pact. On a smaller scale, there is a history of budget forecasts which are distorted for political reasons, especially prior to elections. There are at least two motives for such forecast manipulations: (i) the government is overly optimistic (for instance, overestimates revenues) in order to have more room for maneuver prior to an election; (ii) the government is overly pessimistic (i.e. underestimates the budget balance) in order to show its competence by being able to do unexpected expansionary fiscal policies. The former view is supported, for instance, by Boylan [45] with respect to optimistic revenue forecasts in US states from 1988 to 2004; by Heinemann [46] with respect to optimistic deficit forecasts for Germany’s federal budget from 1969 to 2003; and, more recently, by Boukari and Veiga [47] with respect to optimistic budget forecasts (both revenue and expenditures) in France and Portugal from 1998-2015. Brück and Stephan [48] posit that the Stability and Growth Pact may actually have spurred forecast optimism in the eurozone. Jong-A-Pin, Sturm and de Haan [49] find evidence for both motives in an OECD sample from 1997 to 2006. Overall, there is evidence for both views, possibly with more support for the former.

Manipulations based on overly optimistic forecasts prior to upcoming elections create budget and deficit cycles referred to as “electoral forecast cycle[s]” [48]. Boylan [45] argues that such political manipulations lead to deficits prior to elections despite constitutional balanced budget constraints being in place. Based on a panel data analysis for all 50 US states he provides evidence for pre-election revenue forecasts which are based on favourable estimates of the macroeconomic environment and unrealistic growth assumptions. Table 2 summarises a representative finding of his (while ignoring details, alternative regressions, and sensitivity analyses). The table depicts only some key variables which also happen to be significant at the 5% level (marked by **). It shows that the state government’s forecast error for revenue growth can largely be explained by macroeconomic variables (unemployment and income growth) as well as the forecasting error for personal income. However, state revenue growth forecasts go up, on average, by an additional 2.2% when the fiscal year starts (in most states on 1 July) in the year of the elections (in November). Boylan’s [45] analysis also reveals significant coefficients for underreporting of pre-election state deficit figures, irrespective of the stringency of their constitutional balanced budget constraint.

**Table 2:** Forecast manipulation.

Dependent variable:	State revenue growth forecast error = Forecast state revenue growth -State revenue growth
Fiscal year starts during election year	2.205 (.654)**
State personal income forecast error	1.466 (.257)**
Unemployment	3.433 (.515)**
Income growth	.631 (.105)**
Observations	254
R2	.722

Notes: The table summarises Boylan (p. 420) [45], Table 3, column 4 (which is based on 45 states and the period 1997-2004). Only regressors that are significant at the 5% level (**) are listed. “The regressions include state-fixed effects. Heteroscedastic-consistent standard errors that account for clustering at the state-level are shown in parenthesis”.

In the following model, the incumbent government faces a constitutional balanced budget constraint as currently in place in all US states but Vermont, in Swiss cantons, progressively also in German states as well as in many, if not most, local governments around the world. In such a situation, a state (or local) government’s fiscal latitude can only be increased, if revenues are expected to improve. In principle, any government has two options: raising tax rates and/or predicting a higher tax base. The major drawback of the former is that tax rate increases cannot be concealed and that they are not popular with most electorates in the US and elsewhere, especially prior to elections. The advantage of the latter is that forecasts of higher tax revenues do not receive so much attention, especially on the state level (and if they do, higher tax revenues are not seen as a negative signal). The government can exert a hidden effort by using the additional receipts for providing additional transfers which unsophisticated voters perceive as improvement of their individual economic conditions. This is the scenario discussed in this paper. (Ignoring tax rate increases could also be justified by making a formal argument as in Shi and Svensson [31]. They obtain the optimal tax rate for the “equilibrium without elections” and then use backward induction in the 2-period election cycle to argue that the very same tax rate remains optimal. Moreover, territorial subdivisions may have limited or no influence on the determination of tax rates as in most municipalities, many provinces or regions and, for instance, the German states).

In the model, the prediction of a higher tax base is captured by forecasting a higher growth rate of the economy. Boylan (p. 414) [45] thinks there is a lot of scope for manipulation at the state level: “the executive is likely to have the upper hand in determining the forecasts … in 44 states the governor directly appoints the budget director.” In the following, it is, therefore, assumed that the government can not only influence, but even control the growth forecast made by the budget office. In the model, the growth forecast by the state is measured as deviation from national growth (normalised to zero for simplicity). So, such a *state-specific growth forecast* (henceforth sometimes simply *growth forecast*) can be used as the government instrument for expanding the state’s fiscal latitude and, ultimately, for affecting the incumbent’s re-election chances. Specifically, the government will use its fiscal latitude for increasing *state-specific transfers* (henceforth, synonymously, also *(additional) state transfers*). The focus on transfers is motivated by the empirical evidence (see methods section); but it also allows for a relatively simple model structure.

## An electoral forecast cycle model

Every alternate period an incumbent politician and a challenger representing two different parties run for office. Politicians (henceforth also policymakers) are purely opportunistic and want to convince the voters of being more competent than their opponent. The reason is that voters (henceforth also individuals or agents) are prospective, i.e. they vote for the policymaker who, they think, will be more competent after the elections. Voters’ utility hinges on economic considerations, but also on a more or less strong personal predisposition or sympathy for one of the candidates. This gives rise to an important trade-off; a voter with sympathies for one party may vote for the other, if that party is expected to bring about better economic outcomes for the voter after the elections.

### Preferences, fiscal policy and competence

The utility function for any voter *i* reflects both economic and non-economic components:

$$U_{t}^{i}=\sum_{s=t}^{\infty }{\left(\beta^{i}\right)}^{s-t}E_{s}\left[c_{s}+\alpha \theta^{i}z_{s}\right].$$ (1)

The economic component *c*_*s*_ (consumption) and the sympathy component *θ*^*i*^*z*_*s*_ are additively-separable with relative weight *α* in each period. Discounting between periods with discount factor *β*^*i*^ can ultimately be ignored (as will be seen later); it does not contribute to substance nor exposition. To keep the model tractable, another simplification is that utility is linear in consumption (which has been used before, when it does not affect the key mechanism [50]; alternatively, private and public goods consumption are assumed to be additively-separable and a constant marginal utility of public goods is imposed [31].) Utility derived from sympathy is constrained to $\theta^{i}z_{s}\in \left[-\frac{1}{2},\frac{1}{2}\right]$ since *z*_*t*_ is either $-\frac{1}{2}$ (when party *a* is elected) or $+\frac{1}{2}$ (when party *b* is elected); and the personal sympathy parameter *θ*^*i*^ is uniformly distributed over the interval [−1, 1]. If individual *i* has somewhat more sympathies for party *a*, say at $\theta^{i}=-\frac{1}{2}$, then her utility derived from sympathy is positive ($\frac{1}{4}$), if party *a* is elected ($z_{i}=-\frac{1}{2}$); but it is negative ($-\frac{1}{4}$), if party *b* is elected ($z_{i}=\frac{1}{2}$). The sympathy component represents any attribute of the candidates that does not affect economic policies, be it their stance on societal issues or their good looks. (Without limiting the generality of the analysis we associate the incumbent with party *a* and the challenger with party *b*).

Both politicians *j* = *a*, *b* face a utility function similar to the one for voters consisting, again, of an economic and, if the politician is in power, a non-economic component. The non-economic component is, however, different and includes both a political rent and a political (reputation) cost:

$$\begin{array}{ccc} V_{t}^{j}=\sum_{s=t}^{\infty }V_{s}^{j}=\sum_{s=t}^{\infty }{\left(\beta^{j}\right)}^{s-t} & E_{s} & [c_{s}+{\text{I}}_{s}X_{s}-{\text{I}}_{s-1}{\text{I}}_{s}\xi_{s}D_{s-1}^{2}];\;\;j=a,b;\\ & {\text{I}}_{r} & =\{\begin{array}{c} 1\;\text{if}\;\text{in}\;\text{power}\;\text{in}\;\text{period}\;r;\\ 0\;\text{otherwise}. \end{array} \end{array}$$ (2)

Both policymakers are concerned with consumption. In addition, politician *a* (in power) receives ego rent *X*_*s*_ and bears reputation costs ($\xi_{s}D_{s-1}^{2}$), if she was also in power in the previous period. The reputation costs rise overproportionally (squared) with the previous period violation (*D*_*s*−1_ > 0) of the constitutional balanced budget constraint (requiring *D*_*s*−1_ ≤ 0). The quadratic form is the simplest way of capturing how the government’s trustworthiness and credibility are affected. The legislature and social groups the government has to deal with may “tolerate” small, but not large, deviations and dislike both surpluses and deficits. Again, discounting does not matter for the results.

Voters’ and politicians’ period *t* consumption *c*_*t*_ alike is constrained by nation-wide (per person) income *y*_*t*_; each agent’s *additional* net-of-tax income (1 − *τ*)*ϵ*_*t*_*y*_*t*_ due to state-specific growth rate shock *ϵ*_*t*_; and period-specific *additional* state transfers $T_{t}^{s+}$:

$$c_{t}=y_{t}+\left(1-\tau \right)\epsilon_{t}y_{t}+T_{t}^{s+}.$$ (3)

Tax rate *τ* is taken to be constant and (per person) national income *y*_*t*_ may vary over time, but they are both exogenous. All voters observe the fluctuations of national average income *y*_*t*_, but not the state-specific deviation. State-specific growth rate shock *ϵ*_*t*_ is a random variable with mean *E*_*t*_[*ϵ*_*t*_] = 0 and variance *σ*^2^. $T_{t}^{s+}$ may be interpreted as transfers facilitated by the state government on top of what is normally provided. (Arguing along these lines, Eq 3 could also be obtained by assuming that the normal level of transfers matches the taxes on nation-wide (per person) income *y*_*t*_ as follows: $c_{t}=\left(1-\tau \right)\left(1+\epsilon_{t}\right)y_{t}+T_{t}^{all}$; with $T_{t}^{all}=T_{t}^{normal}+T_{t}^{s+}$ and $T_{t}^{normal}=\tau y_{t}$. Note that deadweight loss and distributional effects are ignored).

Additional state-specific transfers $T_{t}^{s+}$ depend upon fiscal latitude *L*_*t*_ minus the repayment for last period’s deficit (1 − *r*)(*D*_*t*−1_), modulo the government’s positive or negative competence shock, $\eta_{t}^{j}$.

$$T_{t}^{s+}=L_{t}-\left(1+r_{t-1}\right)D_{t-1}+\eta_{t}^{j};$$ (4)

$$L_{t}=\tau \epsilon_{t}^{a}y_{t}.$$ (5)

Fiscal latitude is determined by the state government’s forecast $\epsilon_{t}^{a}$ of state-specific growth rate shock *ϵ*_*t*_. The variable $\epsilon_{t}^{a}$ is incumbent *a*’s instrument and forms the basis for her budget calculations. National interest rate *r*_*t*−1_, known by everybody, determines the repayment costs for any level *D*_*t*−1_. If we think of states without large debt due to the constitutional balanced budget constraint, the rate at which the state government can borrow money should not carry a state-specific risk premium. Hence the interest rate is assumed to be exogenous, though not necessarily constant across periods.

Competence could be interpreted, for instance, as tax collection efficiency or transfer allocation efficiency. Government *j*’s competence $\eta_{t}^{j}$ consists of a skills shock for the current period and another one for the previous period:

$$\eta_{t}^{j}=\mu_{t}^{j}+\mu_{t-1}^{j}.$$ (6)

Hence competence persistence is modelled as an MA(1) process. Limited persistence is a compromise. It allows some persistence while acknowledging that competence also changes over time as new tasks for politicians emerge. For persistence longer than 1 period, the model would not be easily solvable. Rogoff and Sibert’s [41] and Rogoff’s [42] suggestion of an MA(1) process is one of two conditions (the other being the aforementioned assumption of debt being costly) for splitting the model into separate 2-period cycles as is common in this literature. Each cycle consists of an election period and an off-election period. The timing of events (page 11) and the role of these assumptions is outlined further down.

Each skills shock $\mu_{t}^{j}$ is a random variable with mean 0, distribution function $F\left(\mu_{t}^{j}\right)=F\left(•\right)$ and density function $f\left(\mu_{t}^{j}\right)=f\left(•\right)=F^{\prime }\left(•\right)$ which is (weakly) monotonously increasing up to the mean. (For more unusual density functions (for instance, with $F^{\prime \prime }\left(\mu_{t}^{a}\right)<0$ for some $\mu_{t}^{a}\leq 0$), we could get ambiguous results; however, the limiting case of $F^{\prime \prime }\left(\mu_{t}^{a}\right)=0$ for some $\mu_{t}^{a}\leq 0$ or even over the entire range (uniform distribution) is acceptable.) Past shocks are common knowledge, but current or future shocks are unknown to both policymakers and private agents. Even the incumbent does not know her own current competence—an idea suggested by Shi and Svensson [31] – because she always faces new tasks and challenges (like the financial or migration crises) or wants to start new programmes and cannot foresee how efficiently she can manage them. Not knowing her own competence, any incumbent has an incentive to provide additional state transfers in order to appear more competent and increase her re-election chances. Since policymakers do not have an informational advantage, there is no signalling, only moral hazard.

Additional state-specific transfers are actually deficit-financed intertemporal transfers, not income redistribution. State deficits are constitutionally prohibited, but can (and typically will) appear because the incumbent has an incentive to increase her fiscal latitude by raising her state-specific growth forecast above the state-specific growth rate shock (honestly) expected by herself. If the realised state-specific growth rate shock turns out to be much greater than expected, there may be a surplus instead of a deficit. This can be seen from the government budget constraint which is obtained residually; the realised deficit (not expected deficit) is defined as realised government expenditures minus realised government revenues. In our model, this corresponds to realised state-specific transfers $T_{t}^{s+}$ according to Eqs 4 and 5, but excluding the competence shock (which, if positive, only increases transfers costlessly), minus realised tax revenues (*τϵ*_*t*_ *y*_*t*_):

$$\begin{array}{ccc} D_{t} & \equiv & \tau \epsilon_{t}^{a}y_{t}-\left(1+r_{t-1}\right)\left(D_{t-1}\right)-\tau \epsilon_{t}y_{t}\\ & = & \tau \left(\epsilon_{t}^{a}-\epsilon_{t}\right)y_{t}-\left(1+r_{t-1}\right)\left(D_{t-1}\right). \end{array}$$ (7)

The deficit expected by government *a* in election period *t* is:

$$\begin{array}{c} E_{t}^{a}\left[D_{t}\right]=\tau \left(\epsilon_{t}^{a}-E_{t}^{a}\left[\epsilon_{t}\right]\right)y_{t}-\left(1+r_{t-1}\right)\left(D_{t-1}\right). \end{array}$$ (8)

With her state-specific growth forecast $\epsilon_{t}^{a}$ incumbent *a* hopes to facilitate additional state transfers (Eqs 4 and 5), thereby accepting (and expecting) a positive deficit. This is so, when the government has average growth expectations for the state, i.e. $E_{t}^{a}\left[\epsilon_{t}\right]=E_{t}\left[\epsilon_{t}\right]=0$. However, later on we relax this assumption. Proposition 4 considers a perturbation of $E_{t}^{a}\left[\epsilon_{t}\right]$. – Note that state-specific *growth rate shocks* appear in three different forms, their *forecasts* also in three forms. *Growth rate shock* *ϵ*_*t*_ is a random variable with expectational value *E*_*t*_[*ϵ*_*t*_]; (honest) expectations by the incumbent $E_{t}^{a}\left[\epsilon_{t}\right]$ may differ. *Growth forecast* $\epsilon_{t}^{a}$ refers to the manipulated forecast used by the government to justify its fiscal policy choices. Unsophisticated voters’ perception of the state government’s forecast is denoted by $E_{t}^{unsoph}\left[\epsilon_{t}^{a}\right]={\hat{\epsilon_{t}^{a}}}^{unsoph}$; the government’s expectation thereof is $E_{t}^{a}\left[{\hat{\epsilon_{t}^{a}}}^{unsoph}\right]$. See also Eqs 15 to 18 further down.

### Timing of events

The timing of events is summarised in Table 3. In election period *t*, everybody observes last period’s deficit *D*_*t*−1_ and past skills shock $\mu_{t-1}^{a}$. On this basis, incumbent *a* chooses state-specific growth forecast $\epsilon_{t}^{a}$, thus determining her fiscal latitude *L*_*t*_ and providing additional state transfers $T_{t}^{s+}$ for the public according to Eqs 5 and 4, respectively. All voting individuals observe $T_{t}^{s+}$, but only informed sophisticated voters can also observe and make use of the state government’s policy choice of state-specific growth forecast $\epsilon_{t}^{a}$. They can, therefore, deduce current skills $\mu_{t}^{a}$, thereby extracting information about the future competence of the incumbent (since $\eta_{t+1}^{a}=\mu_{t+1}^{a}+\mu_{t}^{a}$). Uninformed sophisticated voters form rational expectations about the incumbent’s choice of forecast manipulation $\epsilon_{t}^{a}$, hence also about $\mu_{t}^{a}$. The description of uninformed sophisticated and unsophisticated voters in Table 3 is similar, but they are conceptually very different. Unsophisticated voters also form expectations about $\mu_{t}^{a}$, but their expectations are based on their perception of the government growth forecast, ${\hat{\epsilon_{t}^{a}}}^{unsoph}$, which hinges on suspicions that can not or, at best, only partially be influenced by the government and its choice of $\epsilon_{t}^{a}$, i.e. they are not (fully) rationally expected. Finally, all voters cast their votes based on their different information sets and their different beliefs of $\mu_{t}^{a}$. What matters is that a share of voters is uninformed (and sophisticated) and a share is unsophisticated. If government policy could be properly judged by all voters, the government would gain nothing from manipulating the forecast and from expanding state-specific transfers.

**Table 3:** The timing of events.

**All voters and incumbent** *a* **observe**: last period’s deficit *D*_*t*−1_ the incumbent’s last period skills $\mu_{t-1}^{a}$ **Incumbent** *a*: chooses state-specific growth forecast $\epsilon_{t}^{a}$ and provides additional state-specific transfers $T_{t}^{s+}$	**All voters observe**: additional state-specific transfers $T_{t}^{s+}$ **Informed sophisticated voters observe**: the incumbent’s state-specific growth forecast $\epsilon_{t}^{a}$	**Informed sophisticated voters**: deduce the incumbent’s current skills $\mu_{t}^{a}$ and vote. **Uninformed sophisticated voters**: form expectations of the incumbent’s current period skills ${\hat{\mu_{t}^{a}}}^{uninf}$ (based on rational expectations of the incumbent’s state-specific growth forecast ${\hat{\epsilon_{t}^{a}}}^{uninf}$) and vote. **Unsophisticated voters**: form expectations of the incumbent’s current period skills ${\hat{\mu_{t}^{a}}}^{unsoph}$ (based on their suspicions of the incumbent’s state-specific growth forecast ${\hat{\epsilon_{t}^{a}}}^{unsoph}$) and vote.	The winner of the period t elections takes office and receives an ego rent. If the incumbent stays in office, she suffers a reputation loss, if the balanced budget constraint was violated in period t. The winner repays the deficit of the previous year.
**Period t**			**Period t+1**

In period (*t* + 1), the winner (incumbent or challenger) takes office and receives an ego rent. If the incumbent stays in office, she also suffers a reputation loss amounting to disutility $\xi_{t}D_{t-1}^{2}$ for having violated the balanced budget constraint. (A government found to have cheated may be in a weaker position in negotiations with the legislature and social groups.) However, voters are no longer relevant for the policymaker’s decision making in (*t* + 1) because they cannot vote in period (*t* + 1). State politicians have no incentive for manipulating their growth estimate $\epsilon_{t}^{a}$. They want to repay the previous period deficit because the deficit is costly (interest and reputation loss) and voters cannot sanction the policymaker for reducing state-specific transfers. That means effectively levying additional taxes to finance the deficit repayment. Given that voters are only concerned with politicians’ competence after the election it does not matter that individuals anticipate in election period *t* that any politician will repay the deficit in the off-election period (*t* + 1). Note also that voters do not consider expected utility in (*t* + 2) in their voting decision in *t*, because even informed sophisticated voters cannot distinguish between the incumbent and her challenger in (*t* + 2) (competence is an MA(1) process only). Politicians, too, are not concerned with the more distant future, because they have no instrument for affecting their own utility or re-election chances in (*t* + 2). The model can, therefore, be split in 2-period cycles consisting of an election period (period *t*) and an off-election period (period *t* + 1).

### The general solution

The incumbent maximises her expected utility in *t* and *t* + 1 (whereby the discount rate is set to 0 for simplicity). The latter depends on the probability of the incumbent of winning the election. **First**, we must, therefore, determine the probability that an individual agent votes for incumbent *a*. It depends on whether a voter expects the incumbent or the challenger to deliver a higher level of utility after the elections, i.e. in *t* + 1. This depends on two components: (i) on the individual’s sympathy *θ*^*i*^ towards the candidates; and (ii) on who can deliver more state-specific transfers which, in turn, depends on the politicians’ skills shock in periods *t* and *t* + 1. Agents do not know future skills of incumbent or challenger; nor can they observe any skills of the challenger in period *t*. However, they may have expectations on the incumbent’s skills ($E_{t}\left[\mu_{t}^{a}\right]$), for instance based on her performance in office in period *t*. It is shown in the S1 Appendix that an individual agent votes for incumbent *a*, if the following inequality (which corresponds to Eq A.6 in Appendix A in the S1 Appendix) holds:

$$E_{t}\left[\mu_{t}^{a}\right]\;>\;\alpha \theta^{i}.$$ (9)

Even if incumbent *a* is expected to be (slightly) less skilled than average, i.e. $E_{t}\left[\mu_{t}^{a}\right]<0$, an individual will still vote for incumbent *a*, if the voter is sufficiently sympathetic towards the incumbent (remember that *θ*^*i*^ < 0 indicates sympathy for incumbent *a* and *α* is a positive weight). Conversely, even if a voter is sympathetic towards the challenger (*θ*^*i*^ > 0), the incumbent could still be chosen, if the incumbent is expected to exhibit sufficiently strong (above average) competence.

**Second** and on this basis, we can derive the probability for the incumbent to win the election (same as Eq B.1 in Appendix B in the S1 Appendix):

$$\text{Prob}\{\underset{\text{informed}\;\text{sophisticated}}{\underset{︸}{\left(1-\gamma -\psi \right)\left[\frac{E_{t}^{inf}\left[\mu_{t}^{a}\right]}{2\alpha }+\frac{1}{2}\right]}}+\underset{\text{uninf.}\;\text{sophisticated}}{\underset{︸}{\gamma [\frac{E_{t}^{uninf}\left[\mu_{t}^{a}\right]}{2\alpha }+\frac{1}{2}]}}+\underset{\text{unsophisticated}}{\underset{︸}{\psi [\frac{E_{t}^{unsoph}\left[\mu_{t}^{a}\right]}{2\alpha }+\frac{1}{2}]}}\geq \frac{1}{2}\}$$ (10)

The probability depends on whether informed sophisticated voters (share (1 − *γ* − *ψ*)), uninformed sophisticated voters (share *γ*) and unsophisticated voters (share *ψ*) think that the incumbent’s skills are above average ($E_{t}\left[\mu_{t}^{a}\right]>0$) or not. The difference for informed sophisticated and the other voters occurs because informed sophisticated voters have all the skills and information for deducing $\mu_{t}^{a}$ from the period *t* transfer Eqs (4) and (5); the other voters do not. Uninformed sophisticated and unsophisticated voters do not observe state-specific growth forecast $\epsilon_{t}^{a}$; instead, they have to use their *perception* of the incumbent’s state-specific growth forecast ${\hat{\epsilon_{t}^{a}}}^{uninf}$ and ${\hat{\epsilon_{t}^{a}}}^{unsoph}$, respectively. Hence, their mistake amounts to $\tau \left({\hat{\epsilon_{t}^{a}}}^{uninf}-\epsilon_{t}^{a}\right)y_{t}$ and $\tau \left({\hat{\epsilon_{t}^{a}}}^{unsoph}-\epsilon_{t}^{a}\right)y_{t}$, respectively—as shown in Appendix B in the S1 Appendix. On this basis, we can derive the incumbent’s probability of winning Prob^*win*^ (identical to Eqs B.6 and B.7):

$${\text{Prob}}^{win}=\text{Prob}\{\mu_{t}^{a}\;\geq \;\gamma \tau \left({\hat{\epsilon_{t}^{a}}}^{uninf}-\epsilon_{t}^{a}\right)y_{t}\;+\;\psi \tau \left({\hat{\epsilon_{t}^{a}}}^{unsoph}-\epsilon_{t}^{a}\right)y_{t}\}$$ (11)

$$=1\;-\;F[\gamma \tau \left({\hat{\epsilon_{t}^{a}}}^{uninf}-\epsilon_{t}^{a}\right)y_{t}\;+\;\psi \tau \left({\hat{\epsilon_{t}^{a}}}^{unsoph}-\epsilon_{t}^{a}\right)y_{t}],$$ (12)

where *F*(•) is the distribution function of the skills shock. Eq 11 shows that a sufficient increase in $\epsilon_{t}^{a}$ (forecast manipulation) leads to a perceived expansion of fiscal latitude (${\hat{\epsilon_{t}^{a}}}^{uninf}<\epsilon_{t}^{a}$ and/or ${\hat{\epsilon_{t}^{a}}}^{unsoph}<\epsilon_{t}^{a}$). The incumbent who is ex ante uncertain about her competence will try to push the right hand side in the bracket of Eq 11 below zero in order to increase the chance of appearing competent, i.e. increase the probability of winning the election above 1/2. Note that the aforementioned equations also show that the incumbent can increase her winning probability even *in equilibrium*. Uninformed sophisticated voters are assumed to rationally expect the government manipulations (${\hat{\epsilon_{t}^{a}}}^{uninf}=\epsilon_{t}^{a}$), but not so unsophisticated voters. The expansion of the winning probability would only be *impossible*, if unsophisticated voters, too, could somehow—for some strange reason—fully anticipate the government machinations. Under the unrealistic assumption of rational expectations for *all* voters we would obtain ${\text{Prob}}^{win}=1-F\left[0\right]=\frac{1}{2}$ in equilibrium.

**Third**, we can turn to the incumbent’s decision problem. The incumbent maximises expected utility over any 2-period cycle, i.e. period *t* utility *plus* period (*t* + 1) utility in case of winning the election multiplied by the probability of winning (as determined in Eqs 11 and 12) *plus* period (*t* + 1) utility in case of losing multiplied by the probability of losing:

$$\begin{array}{ccc} max_{\epsilon_{t}^{a}}\;V & = & max_{\epsilon_{t}^{a}}\;V_{t}^{a}+V_{t+1}^{a}=\\ max_{\epsilon_{t}^{a}} & & E_{t}^{a}\left\{y_{t}+\left(1-\tau \right)\epsilon_{t}y_{t}+T_{t}^{s+}+X-\xi D_{t-1}^{2}\right\}\\ & + & E_{t}^{a}\left\{{\text{Prob}}^{win}\left[y_{t+1}+\left(1-\tau \right)\epsilon_{t+1}y_{t+1}+T_{t+1}^{s+}+X-\xi D_{t}^{2}\right]\right\}\\ & + & E_{t}^{a}\left\{\left(1-{\text{Prob}}^{win}\right)\left[y_{t+1}+\left(1-\tau \right)\epsilon_{t+1}y_{t+1}+T_{t+1}^{s+}\right]\right\}. \end{array}$$ (13)

The S1 Appendix offers a more explicit version (Eq C.1) and a simplified version (Eq C.2) of maximisation problem 13.

Having verified the second order conditions we can characterise the government’s optimal choice of its state-specific growth forecast ${\epsilon_{t}^{a}}^{*}$ with the first order condition (FOC). Again, an extended version is presented in Eq C.3 in the S1 Appendix; a condensed version here:

$$-r_{t}\tau y_{t}+\left[F^{\prime }\left(•\right)\left(\gamma +\psi \right)\tau y_{t}\right]\left[X-\xi {\left(E_{t}^{a}D_{t}\right)}^{2}\right]-\left[1-F\left(•\right)\right]\left[2\xi \tau y_{t}\left(E_{t}^{a}D_{t}\right)\right]=0.$$ (14)

The first term, (−*r*_*t*_*τy*), is the marginal direct net effect of the government’s state-specific growth forecast on deficit, which is negative, because deficit including repayment is costly. The growth forecast is optimally chosen by the government, when the negative marginal direct net effect on deficit (first term) equals the net effect on the expected return if the incumbent stays in power (second and third terms). The latter consists of countervailing effects. The second term depicts the positive marginal impact of higher forecasts on the perceived competence of the incumbent and thus on the voting probability of receiving the (given) expected net return ($X-\xi {\left(E_{t}^{a}D_{t}\right)}^{2}$). The third term captures the negative marginal impact of increased forecasts on the punishment for the lost reputation (since deficit will be increasing) given the chance of winning the elections.

**Forth**, we can now ask several questions. Our first set of question: why is the government’s optimal choice of ${\epsilon_{t}^{a}}^{*}$ overly optimistic and what is the effect thereof? The mechanism is similar to the one in Shi and Svensson’s [31] political budget cycle model. There is moral hazard because a hidden effort (deficit in Shi and Svensson; and forecasts here) is used by the government for expanding fiscal latitude and trying to improve re-election chances (without success there, but successfully here; see Proposition 1 in the next section). Our second set of questions: how is the government’s optimal choice of its forecast manipulation affected by exogenous aspects of the model? Some of the (straightforward) perturbation results (reported in Appendix E in the S1 Appendix) are also similar to Shi and Svensson [31]; higher social costs of incurring a deficit reduce the manipulation; higher benefits of being in office increase the manipulation. Other results (Propositions 2 and 3; first part of next section) differ because unsophisticated voters do not vote rationally here—in contrast to the uninformed voters of the Lohmann [40] and Shi and Svensson [31] type. Then, Proposition 4 (second part of next section) evaluates the effect of government recession expectations.

## Results and discussion

The state budget office’s forecast of state-specific growth, $\epsilon_{t}^{a}$, is publicly available. Informed sophisticated voters use the information rationally to infer the current competence of the policymaker. Uninformed sophisticated voters do not observe the information, but rationally expect the incumbent’s optimal forecast choice at the equilibrium (see Eq 15 below) and hence the policymaker’s competence. This is the standard rational expectations assumption, but applied to a setting with both informed and uninformed voters present (similar to Shi and Svensson [31]). In equilibrium, uninformed sophisticated voters rationally anticipate the *correct* growth forecast that informed sophisticated voters could observe in the first place. That is why there are conceptual objections. Grossman [51], for instance, argues that informed and uninformed agents must hold different beliefs in equilibrium. His assertion was made in the context of a financial market model. According to Grossman, informed and uninformed agents can only hold identical beliefs in equilibrium, if there is an observable economic variable, which contains the entire information the uninformed agent could otherwise not have observed. In his model, a price may (or may not) fulfil this role, but here (or in Shi and Svensson’s [31] model) there is no such variable which would allow the uninformed voters to extract the relevant information. – The rational expectations assumption for uninformed sophisticated voters explains why re-election chances cannot be improved without the existence of unsophisticated voters; this applies to [31] and it applies here, when the share of unsopisticated voters *ψ* is set to zero.

### The role of suspicious unsophisticated voters

Unsophisticated voters base their beliefs on their suspicions of government policy. Up to now, we have not discussed in how far their beliefs depend on actual government policy. From the discussion of Eqs 11 and 12 we know, however, that any deviation from rational expectations affects the winning probability which, in turn, raises the government’s desire to manipulate its forecast. Qualitatively, it does not matter, how much the suspicions by unsophisticated voters deviate from rational expectations as long as a part of those suspicions is exogenous to the government manipulation. How and why unsophisticated voters deviate from rational behaviour is not specified and also not needed; possible reasons are given on page 4. In some sense, unsophisticated voters are similar to “quasi-rational agents” as discussed by Russell and Thaler [5] [6]; unsophisticated voters are still maximising, i.e. trying to vote for the candidate who they think will deliver the highest level of utility for them; however, they use a wrong mapping, in particular they are unable to form rational expectations (unlike uninformed sophisticated voters). In contrast to “quasi-rational agents” à la Russell and Thaler, they may, however, also have to base their decisions on a wrong information set.

The simplest way of capturing the idea of unsophisticated voters is by assuming that they base their beliefs entirely on exogenous suspicions *β* which surpass expected (unbiased) growth *E*_*t*_[*ϵ*_*t*_] (see Eq 16 below). (Note that this is not a deviation from the rational expectations outcome of the manipulated government growth forecast ${\epsilon_{t}^{a}}^{*}$, but from the underlying growth expectations *E*_*t*_[*ϵ*_*t*_] = 0). – When the incumbent optimises with respect to her forecast manipulation, she must take the beliefs of non-rational voters into account. The incumbent’s expectation of the unsophisticated voters’ beliefs may be imprecise (parameter *κ* in Eq 17). – We must also consider that the incumbent has (honest) expectations of the growth prospects of the economy which may differ from expected (unbiased) growth *E*_*t*_[*ϵ*_*t*_] by parameter λ (see Eq 18). Here are formal expressions for all these expectations:

$$E_{t}^{uninf}\left[\epsilon_{t}^{a}\right]={\hat{\epsilon_{t}^{a}}}^{uninf}={\epsilon_{t}^{a}}^{*};$$ (15)

$$E_{t}^{unsoph}\left[\epsilon_{t}^{a}\right]={\hat{\epsilon_{t}^{a}}}^{unsoph}=E_{t}\left[\epsilon_{t}\right]+\beta =\beta ;$$ (16)

$$E_{t}^{a}\left[{\hat{\epsilon_{t}^{a}}}^{unsoph}\right]=E_{t}^{unsoph}\left[\epsilon_{t}^{a}\right]+\kappa =\beta +\kappa ;$$ (17)

$$E_{t}^{a}\left[\epsilon_{t}\right]=E_{t}\left[\epsilon_{t}\right]+\lambda =\lambda .$$ (18)

We can now make use of the rational expectations assumption for uninformed sophisticated voters (Eq 15) and the specifications for expectations of unsophisticated voters and the government (Eqs 16 to 18). The explicit version of the FOC incorporating these modifications (given in Eq C.8 in the S1 Appendix) is the basis for the perturbation results presented in Propositions 2, 3 and 4. However, we can also insert Eqs 15 and 16 into Eq 12 in order to determine the equilibrium probability of getting re-elected. This leads to.

**Proposition 1**- Re-election Chances.

*Manipulations by the incumbent are effective in that they increase the incumbent’s vote share (as long as the optimal growth forecast manipulation by the government*, ${\epsilon_{t}^{a}}^{*}$, *exceeds unsophisticated voters’ suspicions β)*.

*Proof*: Simple inspection of Eq 12; see also discussion thereof on page 13.

*Numerical Example*: The unbiased expected growth rate (*E*_*t*_[*ϵ*_*t*_]) is 0%, but unsophisticated voters are suspicious and think the government might benefit from better economic conditions (e.g. *β* = 1%). Suppose also that the incumbent knows this (*κ* = 0%). Then, any (optimal) growth forecast by the government above 1% ($\epsilon_{t}^{a}>1\%$) would allow the government to increase transfers beyond what unsophisticated voters expect and, thereby, expand its winning probability according to Eq 12 (since uninformed sophisticated voters rationally anticipate the optimal forecast so that the first term in the *F* function of 12 drops out).

How much political support increases depends on the magnitude of the wedge between suspicions by unsophisticated voters and the actual degree of the manipulation. The proposition corroborates evidence by Akhmedov and Zhuravskaya [35], Aidt, Veiga and Veiga [43], and de Haan and Klomp [44] who find that politico-economic machinations positively influence re-election chances. Boylan [45] and Aidt, Veiga and Veiga [43] even find evidence for a “close election bias”, i.e. that government manipulations increase, if the election is closely contested. Note that Eq 12 also indicates that the political support could fall below 50%, if $\beta >{\epsilon_{t}^{a}}^{*}$.

Irrespective of the magnitude of *β*, an increase in suspicions always leads to an upward adjustment of the optimal degree of forecast manipulation—as stated in.

**Proposition 2**- Suspicions by Unsophisticated Voters and Government Expectations.

*If the government expects unsophisticated voters to grow more suspicious (at the equilibrium)*, *even if this is unfounded*, *then it is optimal for the government to increase its growth forecast*, *though underproportionally*.

$$0<\frac{\text{d}{\epsilon_{t}^{a}}^{*}}{\text{d}\beta }=\frac{\text{d}{\epsilon_{t}^{a}}^{*}}{\text{d}\kappa }<1.$$

*Proof*: Appendix D in the S1 Appendix.

*Numerical Example*: Take the example from the previous proposition: expected growth rate *E*_*t*_[*ϵ*_*t*_] = 0%, but unsophisticated voters who were not very suspicious originally now become more sceptical towards (average) economic conditions (e.g. *β* changes from 1% to 2%). Again, suppose the incumbent knows this (*κ* = 0%). Now, any (optimal) growth forecast by the government above 2% ($\epsilon_{t}^{a}>2\%$) would allow the government to expand its winning probability according to Eq 12 (given that uninformed sophisticated voters rationally anticipate the optimal forecast). If the incumbent wanted to preserve its winning probability, it would have to raise the growth forecast one-for-one in order to surpass expected transfers by as much as before. However, that would mean incurring a higher deficit which is costly. Hence, the incumbent will increase the forecast somewhat less, thereby trading off debt repayment (and reputation) costs with winning probability. –Any misjudgement by the government (*κ*) has the same effect as suspicions of unsophisticated voters (*β*) since the government optimises based on what it thinks unsophisticated voters belief.

If the state government anticipates or merely believes an exogenous increase in the unsophisticated voters’ perception of the state government’s growth forecast, the government will respond by increasing the deliberate forecast manipulation even further. This is the “> 0” component. Suppose unsophisticated voters perceive a worsening of fiscal or political transparency. How much of it does the government anticipate? If the incumbent realises that unsophisticated voters become more *sceptical* towards the government or merely imagines it, then she will respond by increasing her manipulation. This may explain why politico-economic manipulations are especially common in developing countries and new democracies. The “< 1” indicates, however, that the wedge between government manipulation and suspicion becomes smaller and smaller when unsophisticated voters become more and more suspicious. It becomes more and more costly for the government (in terms of deficit financed transfers) to “outperform” suspicious unsophisticated voters.

The proposition even covers the situation that unsophisticated voters are overdoing their scepticism by expecting a growth forecast manipulation beyond a level of manipulation that is optimal for the government. Even then it would, again, be optimal for the government to extend its manipulation, if suspicions anticipated by the government were increased. Whatever the situation, an increase in the unsophisticated voters’ suspicions of the state government’s growth forecast manipulation (if anticipated by the government) also implies a reduction of electoral support (implication from Proposition 1). (If the government falsely expects an increase in suspicions, the *expected* electoral support goes down and the forecast manipulation goes up, but actual electoral support will increase, not decrease.) The two propositions together explain the government’s rationale for expanding the manipulation. The government reoptimises by accepting more costly deficit in order to prevent a sharp reduction of the probability of re-election.

The next proposition brings out the difference between uninformed sophisticated and unsophisticated voters (and, consequently, another major difference between this paper on the one hand and Lohmann [40] and Shi and Svensson [31] on the other hand).

**Proposition 3**- The Shares of Different Types of Voters.

*An increase in the share of uninformed sophisticated voters (at the equilibrium) leads to an expansion of the government’s optimal choice of growth forecast manipulation*.

*An increase in the share of unsophisticated voters leads to ambiguous results*.

$$\begin{array}{llll} (i)\;\frac{\text{d}\epsilon_{t}^{a}{}^{*}}{\text{d}\gamma }\;>\;0; & & & (ii)\;\frac{\text{d}\epsilon_{t}^{a}{}^{*}}{\text{d}\psi }\;≷\;0. \end{array}$$

*Proof*: Appendix D in the S1 Appendix.

Result (i) seems to be the intuitive result; that more people can be manipulated leads to more manipulation by the government. In a different setting, such an effect is also found by Shi and Svensson [31] (and could also be shown for [40] given the rational expectations logic). Note that this applies to *uninformed sophisticated* voters who rationally expect the government manipulation in equilibrium. (The winning probability cannot be increased with manipulations; Proposition 1 does not apply.) Result (ii) says that the same does *not necessarily* hold for a larger share of *unsophisticated* voters. That the cycle increases with more unsophisticated voters is likely to hold though, if unsophisticated voters are very suspicious of the government (as can be expected in countries with a high degree of uncertainty and intransparency as, for instance, in developing countries or new democracies). Then manipulating is costly (large ex post deficit; deficit costs increase overproportionally with higher deficits) because the forecast manipulation has to be large in order to have at least some effect on the winning probability. Nonetheless, impressing highly suspicious voters is so costly that the wedge between manipulation and suspicions is kept to a minimum. Increasing the share of unsophisticated voters means that more voters can be affected. The manipulation becomes more effective and will be extended despite the high costs for increasing the deficit even further. If unsophisticated voters are, however, not very suspicious, the wedge between government manipulation and suspicions can be larger because manipulation is not very costly. Individual unsophisticated voters are very likely to vote for the incumbent. Hence the government *may* (depending on the properties of *F*(•) and the parameter constellations) even be able to get re-elected when it reduces its manipulation knowing that it can now affect a larger share of all voters very effectively.

### Recession expectations by the government

It is almost commonplace now that “Recession and Re-election Don’t Mix” [52]. The title was chosen by *The New York Times* at the occasion of recession fears prior to the then upcoming re-election campaign of George Bush (sen.) and potential problems for Bush’s advisors to find “ways to boost the economy for their political advantage in an election year”. If all voters fully adjusted their expectations to an upcoming recession, economic downturns would not have a negative effect for re-elections as suggested by *The New York Times*. In reality, it is unlikely that *all* voters adjust their expectations of government policy as soon as state-specific recession tendencies are discussed in the media. In particular, unsophisticated voters should remain unsophisticated, if recession expectations change [17].

In this paper, it is assumed that no unsophisticated voter is sophisticated enough to adjust his/her expectations. The same qualitative result is, however, obtained, if we assume that only a share of *unsophisticated* voters responds to recession expectations and/or unsophisticated voters respond only partially. (Note that the behaviour of *sophisticated* voters is not affected by changes in recession expectations because they are either informed and can infer the government’s skills from observing the government’s growth forecast or they are uninformed and can rationally expect the government’s state-specific growth forecast.) The question is then how an opportunistic government can manipulate policies in the face of its own recession fears knowing that part of the electorate will attribute a bad economy to bad government policies. Boylan [45] suggests that “government officials can avoid choosing between raising taxes and cutting government programs by making optimistic forecasts when the economy is headed for a downturn.” The next proposition shows how the typically optimistic forecasts (based on an—on average—steady economy as discussed before) will be adjusted to cushion the effects of an expected recession.

**Proposition 4**- Recession (or Boom) Expectations.

*Imminent recession expectations by the government* (*lower* $E_{t}^{a}\left[\epsilon_{t}\right]$ *in* Eq 13) *decrease the government’s optimal growth forecast at the equilibrium*, *but underproportionally*. (*Analogously*, *boom expectations increase optimal growth forecasts*, *but*, *again*, *underproportionally*).

$$0<\frac{\text{d}{\epsilon_{t}^{a}}^{*}}{\text{d}\lambda }<1.$$

*Proof*: Appendix D in the S1 Appendix.

*Numerical Example*: Again, take the example from Proposition 1: expected growth rate *E*_*t*_[*ϵ*_*t*_] = 0%; unsophisticated voters are suspicious (*β* = 1%). In addition, the government changes her (honest) growth expectations to, for instance, λ = −1. Consequently, it also thinks (maybe falsely) that it needs a growth forecast of above 2% ($\epsilon_{t}^{a}>2\%$) to surpass transfer expectations by voters. Compared to no (honest) recession expectations the government would want to (optimally) increase the forecast and hence the deficit, but, once again, not one-for-one because it is, again, trading off debt repayment (and reputation) costs with winning probability.

If we perturb the equilibrium so that the policymaker expects a downturn (reduced (honest) expectations by the government of state-specific growth, $E_{t}^{a}\left[\epsilon_{t}\right]<E_{t}\left[\epsilon_{t}\right]=0$), she reduces her state-specific growth forecast $\epsilon_{t}^{a}$, but not as much as $E_{t}^{a}\left[\epsilon_{t}\right]$ goes down. Hence the state government’s growth forecast will be even more optimistic relative to the government’s realistic (honest) growth expectations. This means that the government also *expects* to run an even higher deficit. An anticipated recession leads to an amplified budget cycle. This amounts to a countercyclical policy effect, but for the wrong reasons, namely that an opportunistic incumbent tries to manipulate forecasts and fiscal policies in order to ensure her own re-election. In an empirical study on sub-Saharan African countries, Diallo [53] finds that democratic institutions make fiscal policies countercyclical. He had suspected, but could not find empirically, that political business cycles (caused by political competition) produce procyclical policies. (Procyclical policies in developing countries are, however, found in other papers, for instance [19].) This paper argues that political forecast cycles may contribute to a countercyclical policy effect in election years.

But why will the government adjust as suggested in Proposition 4? Suppose the government did not adjust its state-specific growth forecast $\epsilon_{t}^{a}$, but state growth was indeed lower. Then the deficit would increase, but that would be costly. So it would have been optimal for the government to reduce its growth forecast $\epsilon_{t}^{a}$. However, a reduction of $\epsilon_{t}^{a}$ means accepting lower electoral support from unsophisticated voters (since ${\hat{\epsilon_{t}^{a}}}^{unsoph}$ does not change in Eqs 11 and 12). Thus, the government wants to re-optimise by trading off deficit costs for loss of re-election probability; and it chooses to reduce its growth forecast $\epsilon_{t}^{a}$ only underproportionally in order to preserve a sufficiently positive effect on its re-election chances. (If the expected recessionary shock is too large, not even that may be possible. This would be the case, if the government’s optimal choice turned out to be a negative state-specific growth forecast. This would also imply a negative provision of transfers ($T_{t}^{s+}<0)$).

**Corollary 1**- The Effect on Debt.

*If the imminent recession expectations by the government* (*lower* $E_{t}^{a}\left[\epsilon_{t}\right]$) *are justified*, *the actual deficit increases* (*at the equilibrium*) [*decreases with boom expectations*].

$$\frac{\text{d}D}{\text{d}\lambda }<0.$$

Given that the government responds underproportionally to an expected downturn or upturn (Proposition 4), the corollary follows directly from Eqs 8 and 7. Essentially, the corollary says that political opportunism encourages the government to run more countercyclical policies, at least during election years. Note, however, that the deficit would be reduced (augmented), if the recession (boom) expectations did *not* materialise.

In the real world, the countercyclicality result could be stronger. In this model, a deficit is also costly because of the reputation costs the government suffers from in the post-election period according to Eq 2. However, voters will probably not punish the government so much for having missed the balanced budget requirement ex post, if it turns out that it happened during a recession. As deficits might be less costly in the real world compared to the model world, this would reinforce the incumbent’s willingness to use overly optimistic forecasts for facilitating an expansionary policy during a recession.

It is good news that opportunistic government behaviour may also have a stabilising effect in case of a boom or a recession. It is particularly good news for many developing countries that used to suffer and mostly still suffer from procyclical fiscal policies [19]. At least during a recession occurring in an election year, the economic contraction would be somewhat alleviated according to Proposition 4. This result goes beyond the mere existence of political forecast and budget cycles (for the standard case of no recession expectations) as confirmed by empirical evidence ([45] and others) and the theoretical result of this paper. In addition, it says that expected recessions *increase* such a political forecast and budget cycle. This is a new theoretical finding, which can, in principle, be tested empirically.

To my knowledge, the effect of *expected recessions* on political cycles has been ignored in the literature thus far. Boylan [45] tries to capture the effect of *actual recessions* on forecasts in US states by including changes in *actual* unemployment, income and inflation, but cannot test, if recessionary *a priori expectations* affect the magnitude of forecast manipulations. Kamlet, Mowery and Su [54] find that *actual recessions* affect long-run, but not short-run forecasts. Furthermore, their results are based on federal US data (where only a soft fiscal limit applies) and not on data for US states (where constitutional balanced budget constraints are in place). It may not be straightforward to extract data for true expectations by state governments on their economic outlook, because the data *published* by the states is exactly what we suspect, namely, intentionally, too optimistic. It is not straightforward to overcome this problem. In the US context, we might be able to use the national economic outlook (which is known to be less biased) and break it down to the state level. This would, however, require the use of specific state level economic indicators as well as some understanding of the state level dynamics of US business cycles (see, for instance, [55]). On this basis, it might be possible to construct a panel data set with US state governments’ (honest) recession expectations.

As for other countries, one might be able to use the national forecasts as proxies for (honest) regional or state-level government expectations, if sub-national units are not too heterogeneous. On a national level, for instance for developing countries, one could use *International Financial Statistics*/*World Economic Outlook* forecasts for the election year published by the International Monetary Fund in the year before. This is the recession/boom information available to the government when it has to form its own (honest) expectations prior to taking decisions on politico-economic manipulations.

## Conclusion

The paper argues that much of the empirical evidence on political cycles falls into place, if we introduce suspicious unsophisticated voters into the theoretical political cycle literature. In particular, it can be shown (to my knowledge for the first time in a theoretical model) that the government actually succeeds in *systematically* boosting its re-election chances. This is confirmed by empirical studies [44] [43] [35] which argue that the incumbent’s vote share increases with electoral manipulations conducted by the government.

The paper posits that part of the electorate has suspicions towards the government which are “context-specific” and increase with uncertainties and any type of intransparency present in a country’s economic and political system. Uncertainties and intransparencies could be present in a country’s fiscal constitution, the functioning of the political debate, the running of the political process in parliament, the power structure within the government, the electoral system, the distrust of politicians in some new democracies, or the high levels of corruption in some developing countries. Unsophisticated voters are prospective, i.e. interested in future outcomes which depend on the government’s abilities or competence; but they are not able to (fully) rationally expect the government’s machinations (a partial response to actual current or expected future government behaviour may be possible though). In this paper, it can be shown that an increase in suspicions by part of the electorate increases the government’s willingness to manipulate. The reason is that the government will try to “outperform” suspicious unsophisticated voters. It will try to appear more competent than expected by those voters in order to increase its chance of being re-elected. These results and the aforementioned considerations suggest the following logic which is straightforward, but has been ignored thus far: politically motivated manipulations increase with the degree of suspicions held by unsophisticated voters; suspicions originate in uncertainties and intransparencies and are particularly relevant in the aforementioned “contexts”, in particular in developing countries and new democracies; it is not surprising, therefore, that, in empirical research, political cycles are especially found in specific “contexts”.

The model presented in this paper also makes a new prediction which could be tested empirically. It is in the nature of an opportunistic policymaker that she will adjust her manipulations to her own expectations of the conditions in the economy given the suspicions of unsophisticated voters. If an incumbent expects an imminent downturn, she may reduce her otherwise envisaged forecast (to avoid excessive deficit costs), but not by as much as her expectations have gone down because she tries to avoid or limit increases in taxes or cuts in spending programmes just before the election. Hence she may be keen on producing particularly optimistic forecasts (relative to her own honest expectations) in order not to appear incompetent and loose too many votes. If there are boom expectations, forecast optimism may be dampened. Overall, political opportunism thus produces, unintentionally, a countercyclical policy effect. Despite having clear empirical implications this finding has not been discussed in the literature thus far (apart from the hunch by Boylan [45] mentioned in the main text).

The paper also offers a theoretical explanation for political distortions found in forecasts by US states [45], where most budget directors are directly appointed by the governor. Based on overly optimistic revenue forecasts the incumbent state government can conduct expansionary fiscal policies in order to appear more competent prior to an upcoming election. Since the resulting deficit can only be observed afterwards, the government can effectively circumvent a constitutional balanced budget constraint. As a result, there are political forecast and budget cycles in the state. More generally, however, these findings may also apply to European countries where *(constitutional) balanced budget constraints* are or will be in place. Constitutional debt brakes in Switzerland (from 2003) and Germany (from 2016 nationally, from 2020 on the state (Länder) level), for instance, focus on the deficit; those in Poland, Spain, Italy and Austria, for instance, on the 60% debt-to-GDP ratio; the European Fiscal Compact as of 2013 refers to both deficit and debt. The results may also relate to politically motivated manipulations by governments vis-à-vis their European partners as, for instance, when Greece obtained membership of the Euro area on the basis of fudged budget figures.

It is somewhat disturbing that budget manipulations can be successful despite national (or state level) constitutional balanced budget constraints and international rules and oversight. This paper pinpoints a possible underlying mechanism: the government tries to “outperform” suspicious unsophisticated voters. (Obviously, the same mechanism could also apply in other settings, not least the corporate world.) It is hard to think of remedies. How could political decision making be improved given this strong underlying mechanism? As for policy analysis, it is important to take government manipulations as a prevalent feature into account. The good news is that political opportunism produces countercyclical fiscal policies in election years.

Further theoretical research may be devoted to adjusting the model to a situation in which the government does not have direct control over fiscal forecasts—as in the European context. As for empirical research, one could try to verify or reject the theoretical prediction that fiscal policy turns more countercyclical in election years due to political opportunism. In the context of developing countries, it would be interesting to see which role the presumed countercyclicality effect plays in alleviating their typical procyclicality problem. Understanding the key mechanisms against the backdrop of US states may have been a first step.

## Supporting information

S1 Appendix (PDF)
