# Supplementary material for: Political cycles: Beyond rational expectations
Source: PLoS One. 2018 Oct 11;13(10):e0203390. doi: 10.1371/journal.pone.0203390 (PMC6181349; doi:10.1371/journal.pone.0203390)
Supplement: S1 Appendix — (PDF) [file pone.0203390.s001.pdf]

S1 Appendix The appendix presents indications for the general solution in the model section and for the derivation of the propositions in the results section.

A Probability of an individual to vote for the incumbent

First, we consider an individual voter, no matter if sophisticated (informed or uninformed) or unsophisticated. She votes for incumbent a, if

$$\underbrace{E_t[c^a_{t+1} + \alpha\theta^i(-\frac{1}{2})]}_{\text{exp. utility when } a \text{ in power}} \quad > \quad \underbrace{E_t[c^b_{t+1} + \alpha\theta^i(+\frac{1}{2})]}_{\text{exp. utility when } b \text{ in power}} \quad . \tag{A.1}$$

Depending on who is in power, $t+1$ consumption will typically differ because of differences in policymakers' competence and individuals' expectations about it:

$$E_t[c^j_{t+1}] = E_t[y_t] + E_t[(1-\tau)\epsilon^j_{t+1}y_{t+1}] + E_t[T^{s+^j}_{t+1}], \qquad j = a, b; \tag{A.2}$$

$$E_t[T^{s+^j}_{t+1}] = E_t[\tau\epsilon^j_{t+1}y_{t+1}] - (1+r_t)D_t + E_t[\eta^j_{t+1}], \qquad j = a, b. \tag{A.3}$$

Eq A.3 says that the period t deficit must be repaid in period $(t+1)$. However, the policymaker will try not to borrow in period $(t+1)$ because there is no election at the end of that period and a non-balanced budget is costly (repayment and reputation costs; nonetheless, any policymaker will probably end up with a deficit or surplus in $(t+1)$ because actual growth in Eq 7 is a random variable; see also the discussion of the timing of events on page 11). The best growth forecast for the incumbent in $(t+1)$ is, therefore, $\epsilon^j_{t+1} = E[\epsilon_{t+1}] = 0$. As a result, state-specific transfers in $(t+1)$ are bound to be negative (i.e. taxes) corresponding to deficit repayment modulo the effect of the policymaker's competence. Individuals have no idea about the skills shock of either potential policymaker in $t+1$. Nor do they know the skills shock of the challenger in period t, and, therefore, expect 0. However, they can use the incumbent's period t transfer policy to draw conclusions about her skills shock in period t (not shown here, but further down; the difference between informed and uninformed sophisticated as well as unsophisticated voters will then be exploited). We obtain

$$E_t[T^{s+^b}_{t+1}] = -E_t[(1+r_t)D_t]; \tag{A.4}$$

$$E_t[T^{s+^a}_{t+1}] = -E_t[(1+r_t)D_t] + E_t[\mu^a_t], \tag{A.5}$$

where D_t denotes the deficit which depends, according to Eq 7, on the difference of realised state-specific growth ϵ_t and the incumbent's optimal choice for the period t state-specific growth forecast ϵ^{a*}_t (to be determined further down). Combining Eqs A.1 to A.5 we can obtain a condition for an individual to vote for incumbent a (which corresponds to condition 9 in the main text):

$$E_t[\mu^a_t] \quad > \quad \alpha\theta^i. \tag{A.6}$$

Using the distribution of the skills shock we can determine the probability (Pr) of any voter, be she sophisticated (informed or uninformed) or unsophisticated, to vote for incumbent a:

$$Pr[E_t[\mu^a_t] - \alpha\theta^i \geq 0] = \frac{E_t[\mu^a_t] - (-\alpha)}{\alpha - (-\alpha)} = \frac{E_t[\mu^a_t]}{2\alpha} + \frac{1}{2}. \tag{A.7}$$

B Probability of the incumbent to win

Now, we can determine the probability $Prob$ that incumbent a obtains 50% of the votes in the period t elections. It is the probability that all shares of voters times their

individual probability Pr to vote for incumbent a (as determined in Eq A.7) is greater or equal to $\frac{1}{2}$. However, the individual probability Pr is different for informed sophisticated, uninformed sophisticated and unsophisticated voters because their expectations of period t skills, $E_t[\mu_t^a]$, differ. The probability for the incumbent to win the election – Eq 10 in the main text – is repeated here:

$$\text{Prob}\left\{\underbrace{(1-\gamma-\psi)\,[\frac{E_t^{inf}[\mu_t^a]}{2\alpha}+\frac{1}{2}]}_{\text{informed sophisticated}}+\underbrace{\gamma\,[\frac{E_t^{uninf}[\mu_t^a]}{2\alpha}+\frac{1}{2}]}_{\text{uninformed sophisticated}}+\underbrace{\psi\,[\frac{E_t^{unsoph}[\mu_t^a]}{2\alpha}+\frac{1}{2}]}_{\text{unsophisticated}}\geq\frac{1}{2}\right\}$$

So why is there a difference in expectations for the three types of voters? Rewrite the government transfer Eqs 4 and 5 for period t:

$$T_t^{s+}=\tau\epsilon_t^a y_t-(1+r_{t-1})D_{t-1}+\mu_t^a+\mu_{t-1}^a. \tag{B.2}$$

For informed sophisticated voters we obtain:

$$E_t^{inf}[\mu_t^a]=\mu_t^a=T_t^{s+}-\tau\epsilon_t^a y_t+(1+r_{t-1})D_{t-1}-\mu_{t-1}^a. \tag{B.3}$$

State-specific transfers T_t^{s+}, previous period deficit D_{t-1}, previous period competence μ_{t-1}^a, national (per person) income y_t, interest rate r_{t-1} and the tax rate τ can be observed by everybody. The point is that informed sophisticated voters can determine $E_t^{soph}[\mu_t^a]$ deterministically, because they can also observe the incumbent's state-specific growth forecast, ϵ_t^a. By contrast, uninformed sophisticated voters must form an estimate of the incumbent's skills, $\widehat{\mu_t^a}^{uninf}$, based on their *perception* of the state government's growth forecast $\widehat{\epsilon_t^a}^{uninf}$:

$$\begin{aligned}E_t^{uninf}[\mu_t^a]=\widehat{\mu_t^a}^{uninf} &= T_t^{s+}-\tau\widehat{\epsilon_t^a}^{uninf}y_t+(1+r_{t-1})D_{t-1}-\mu_{t-1}^a\\ &= \underbrace{T_t^{s+}-\tau\epsilon_t^a y_t+(1+r_{t-1})D_{t-1}-\mu_{t-1}^a}_{\mu_t^a\text{ from (B.3)}}+\tau\epsilon_t^a y_t-\tau\widehat{\epsilon_t^a}^{uninf}y_t;\\ E_t^{uninf}[\mu_t^a]=\widehat{\mu_t^a}^{uninf} &= \mu_t^a+\tau(\epsilon_t^a-\widehat{\epsilon_t^a}^{uninf})y_t. \end{aligned}\tag{B.4}$$

Uninformed sophisticated voters overestimate the incumbent's competence by $\tau(\epsilon_t^a-\widehat{\epsilon_t^a}^{uninf})y_t$. By the same logic, unsophisticated voters overestimate the incumbent's competence by $\tau(\epsilon_t^a-\widehat{\epsilon_t^a}^{unsoph})y_t$:

$$E_t^{unsoph}[\mu_t^a]=\widehat{\mu_t^a}^{unsoph}=\mu_t^a+\tau(\epsilon_t^a-\widehat{\epsilon_t^a}^{unsoph})y_t. \tag{B.5}$$

Using Eqs B.3 to B.5 we can now determine the probability Prob^{win} that incumbent a receives 50% of the votes in period t:

$$\begin{aligned}\text{Prob}^{win} &= \text{Prob}\left\{(1-\gamma-\psi)\,[\frac{\mu_t^a}{2\alpha}+\frac{1}{2}]+\gamma\,[\frac{\mu_t^a+\tau(\epsilon_t^a-\widehat{\epsilon_t^a}^{uninf})y_t}{2\alpha}+\frac{1}{2}]\right.\\ &\qquad\left.+\;\psi\,[\frac{\mu_t^a+\tau(\epsilon_t^a-\widehat{\epsilon_t^a}^{unsoph})y_t}{2\alpha}+\frac{1}{2}]\geq\frac{1}{2}\right\};\\ &= \text{Prob}\left\{\frac{\mu_t^a}{2\alpha}+\gamma\,\frac{\tau(\epsilon_t^a-\widehat{\epsilon_t^a}^{uninf})y_t}{2\alpha}+\psi\,\frac{\tau(\epsilon_t^a-\widehat{\epsilon_t^a}^{unsoph})y_t}{2\alpha}+\frac{1}{2}\geq\frac{1}{2}\right\};\\ \text{Prob}^{win} &= \text{Prob}\left\{\mu_t^a\geq\gamma\,\tau(\widehat{\epsilon_t^a}^{uninf}-\epsilon_t^a)y_t+\psi\,\tau(\widehat{\epsilon_t^a}^{unsoph}-\epsilon_t^a)y_t\right\}; &\text{(B.6)}\\ &= 1-F\,[\,\gamma\,\tau(\widehat{\epsilon_t^a}^{uninf}-\epsilon_t^a)y_t+\psi\,\tau(\widehat{\epsilon_t^a}^{unsoph}-\epsilon_t^a)y_t]\,, &\text{(B.7)}\end{aligned}$$

Fig 1. Bell-shaped competence density function F' as an example.

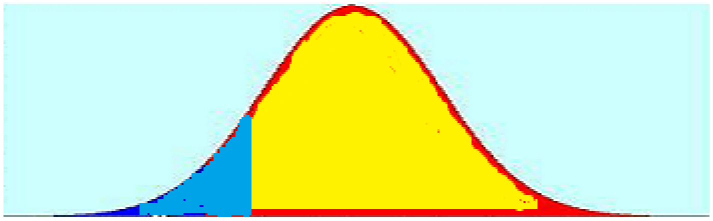

where $F(\bullet)$ is the distribution function of the skills shock.

The marked area towards the right (light grey or yellow [if in colour]) under the density function depicted in the figure corresponds to the probability described by Eq B.6 and by the distribution function representation in Eq B.7. The expected competence overall (combine Eqs B.3 to B.5) is always greater than the actual competence, if the government's state-specific growth estimate perceived by uninformed sophisticated and/or unsophisticated voters is smaller than the actual government growth estimate ($\widehat{\epsilon_t^a}^{uninf} < \epsilon_t^a$ and/or $\widehat{\epsilon_t^a}^{unsoph} < \epsilon_t^a$). Then the probability (see Eq B.7 or the light grey [or yellow] area under the density function) is always greater than $\frac{1}{2}$. We can see that expanding fiscal latitude by choosing an overly optimistic state-specific growth forecast increases the government's chance to be re-elected. A similar effect would occur, if uninformed sophisticated or unsophisticated voters were to reduce their perception of the government growth forecast for some reason.

C The incumbent's maximisation problem

Prior to elections, incumbent a would like to maximise her utility over periods t and $(t+1)$ by choosing ϵ_t^a (see the timing of events on page 11). Period $t+1$ utility is the sum of the utilities for winning and losing the election weighted by the probability determined previously:

$$\begin{aligned}
max_{\epsilon_t^a}\ V \quad &= \quad max_{\epsilon_t^a}\ V_t^a + V_{t+1}^a \quad = \\
max_{\epsilon_t^a} \quad & E_t^a\{y_t + (1-\tau)\epsilon_t y_t + \underbrace{\tau\epsilon_t^a y_t - (1+r_{t-1})D_{t-1} + \eta_t^a}_{T_t^{s+}\text{ (Eqs 4 and 5)}} + X - \xi D_{t-1}^2\} \\
+ \quad & E_t^a\{\underbrace{[1 - F[\gamma\tau(\widehat{\epsilon_t^a}^{uninf} - \epsilon_t^a)y_t \ + \ \psi\tau(\widehat{\epsilon_t^a}^{unsoph} - \epsilon_t^a)y_t]]}_{\text{prob. incumbent wins}} \\
& \qquad [y_{t+1} + (1-\tau)\epsilon_{t+1}y_{t+1} + \underbrace{\tau\epsilon_{t+1}^a y_{t+1} - (1+r_t)D_t + \eta_{t+1}^a}_{T_{t+1}^{s+}} + X - \xi D_t^2]\} \\
+ \quad & E_t^a\{\underbrace{[F[\gamma\tau(\widehat{\epsilon_t^a}^{uninf} - \epsilon_t^a)y_t \ + \ \psi\tau(\widehat{\epsilon_t^a}^{unsoph} - \epsilon_t^a)y_t]]}_{\text{prob. incumbent loses}} \\
& \qquad [y_{t+1} + (1-\tau)\epsilon_{t+1}y_{t+1} + \underbrace{\tau\epsilon_{t+1}^b y_{t+1} - (1+r_t)D_t + \eta_{t+1}^b}_{T_{t+1}^{s+}}]\}. \qquad \text{(C.1)}
\end{aligned}$$

Next, substitute in for the expected current debt ($E_t^a[D_t] = \tau(\epsilon_t^a - E_t^a[\epsilon_t])y_t - (1+r_{t-1})(D_{t-1})$); simplify expectations when they are zero (expectations of the future growth rate shock, $E_t^a[\epsilon_{t+1}] = 0$; and expectations of the optimal future growth rate forecast, $E_t^a[\epsilon_{t+1}^a] = E_t^a[\epsilon_{t+1}^b] = 0$); and acknowledge that the incumbent knows her past, but not her present and future skills, nor the skills shock

of the challenger ($E_t^a[\eta_t^a] = E_t^a[\mu_{t-1}^a] + E_t^a[\mu_t^a] = E_t^a[\mu_{t-1}^a] + 0$; and $E_t^a[\eta_{t+1}^a] = E_t^a[\eta_{t+1}^b] = 0$). Now, the maximisation problem looks as follows:

$$\begin{aligned} max_{\epsilon_t^a} \quad & y_t + y_{t+1} + E_t^a[\epsilon_t]y_t - r_t\tau(\epsilon_t^a - E_t^a[\epsilon_t])y_t + r_t(1+r_{t-1})D_{t-1} + \mu_{t-1}^a + X - \xi D_{t-1}^2 \\ + \quad & [1 - F[\gamma\tau(\widehat{\epsilon_t^a}^{uninf} - \epsilon_t^a)y_t \;+\; \psi\tau(E_t^a[\widehat{\epsilon_t^a}^{unsoph}] - \epsilon_t^a)y_t]] \\ & [X - \xi[\tau(\epsilon_t^a - E_t^a[\epsilon_t])y_t - (1+r_{t-1})D_{t-1}]^2]. \end{aligned} \tag{C.2}$$

The elaborate version of the first order condition (FOC) is given here (and a condensed version as Eq 14 in the main text):

$$\begin{aligned} -r_t\tau y_t \quad + \quad & F'[\gamma\tau(\widehat{\epsilon_t^a}^{uninf} - \epsilon_t^a)y_t \;+\; \psi\tau(E_t^a[\widehat{\epsilon_t^a}^{unsoph}] - \epsilon_t^a)y_t] \\ & (\gamma+\psi)\tau y_t[X - \xi[\tau(\epsilon_t^a - E_t^a[\epsilon_t])y_t - (1+r_{t-1})D_{t-1}]^2] \\ - \quad & [1 - F[\gamma\tau(\widehat{\epsilon_t^a}^{uninf} - \epsilon_t^a)y_t \;+\; \psi\tau(E_t^a[\widehat{\epsilon_t^a}^{unsoph}] - \epsilon_t^a)y_t]] \\ & 2\xi\tau y_t[\tau(\epsilon_t^a - E_t^a[\epsilon_t])y_t - (1+r_{t-1})D_{t-1}] = 0. \end{aligned} \tag{C.3}$$

Having verified the second order condition for a well-behaved maximisation problem the FOC determines the government's optimal state-specific growth forecast ϵ_t^{a*}. To facilitate the analysis, the expectational terms are discussed and formalised in the main text. Expectations of uninformed sophisticated voters were said to be rational; and suspicions of unsophisticated voters exogenous. Furthermore, the government has expectations of those suspicions; and it has expectations of economic growth that may deviate from (unbiased) expected economic growth. The following equations correspond to Eqs 15 to 18 in the main text:

$$E_t^{uninf}[\epsilon_t^a] \;=\; \widehat{\epsilon_t^a}^{uninf} \;=\; \epsilon_t^{a*}; \tag{C.4}$$

$$E_t^{unsoph}[\epsilon_t^a] \;=\; \widehat{\epsilon_t^a}^{unsoph} \;=\; E_t[\epsilon_t] + \beta \;=\; \beta. \tag{C.5}$$

$$E_t^a[\widehat{\epsilon_t^a}^{unsoph}] \;=\; E_t^{unsoph}[\epsilon_t^a] + \kappa \;=\; \beta + \kappa. \tag{C.6}$$

$$E_t^a[\epsilon_t] \;=\; E_t[\epsilon_t] + \lambda \;=\; \lambda. \tag{C.7}$$

The first order condition becomes:

$$\begin{aligned} -r_t\tau y_t \quad + \quad & F'[\psi\tau(\beta + \kappa - \epsilon_t^{a*})y_t] \\ & (\gamma+\psi)\tau y_t[X - \xi[\tau(\epsilon_t^{a*} - \lambda)y_t - (1+r_{t-1})D_{t-1}]^2] \\ - \quad & [1 - F[\psi\tau(\beta + \kappa - \epsilon_t^{a*})y_t]] \\ & 2\xi\tau y_t[\tau(\epsilon_t^{a*} - \lambda)y_t - (1+r_{t-1})D_{t-1}] = 0. \end{aligned} \tag{C.8}$$

Note that the effect of uninformed sophisticated voters on the winning probability is gone with the assumption of rational expectations (see also Eq B.7), but the existence of uninformed sophisticated voters does still create a distortion, namely the increase in the coefficient of the second term ($\gamma + \psi$ instead of only ψ).

D Perturbation results for the propositions

Perturbation results in Appendix E and in the Propositions of the results section are obtained by using the Implicit Function Theorem.

Derivatives with respect to any variable x of the FOC around the optimal value ϵ_t^{a*} will be denoted $\frac{\mathrm{d}\frac{\mathrm{d}V}{\mathrm{d}\epsilon_t^a}|_{\epsilon_t^{a*}}}{\mathrm{d}x} =: V_{\epsilon_t^a x}$. The derivations of the marginal effect of changes in exogenous variables *on* the equilibrium value of the government's optimal choice of the state-specific growth forecast $(\epsilon_t^a)^*$ are given here:

Proposition 2:

$$\frac{\mathrm{d}\epsilon_t^{a*}}{\mathrm{d}E_t^a[\widehat{\mu_t^a}^{uninf}]} = \frac{d\epsilon_t^{a*}}{d\beta} = \frac{d\epsilon_t^{a*}}{d\kappa} \tag{D.1}$$

$$= -\frac{V_{\epsilon_t^a E_t^a[\widehat{\mu_t^a}^{uninf}]}}{V_{\epsilon_t^a \epsilon_t^a}} = -\frac{A+C}{-(A+B+C+D)} < 1 \quad \text{and positive,}$$

where

$$\begin{aligned}
A &= \psi\tau y_t F''[\psi\tau(\beta+\kappa-\epsilon_t^{a*})y_t](\gamma+\psi)\tau y_t[X-\xi(E_t^a D_t)^2] > 0;\\
B &= F'[\psi\tau(\beta+\kappa-\epsilon_t^{a*})y_t](\gamma+\psi)\tau^2 y_t^2 2\xi(E_t^a D_t) > 0;\\
C &= F'[\psi\tau(\beta+\kappa-\epsilon_t^{a*})y_t]\psi\tau^2 y_t^2 2\xi(E_t^a D_t) > 0;\\
D &= [1-[F[\psi\tau(\beta+\kappa-\epsilon_t^{a*})y_t]]\tau^2 y_t^2 2\xi > 0;\\
& \quad \text{with} \quad E_t^a D_t = \tau(\epsilon_t^{a*}-\lambda)y_t - (1+r_{t-1})D_{t-1}\\
& \quad \text{and} \quad X-\xi(E_t^a D_t)^2 > 0 \quad \text{(exp. benefit from being in office).}
\end{aligned}$$

Proposition 3:

$$\frac{\mathrm{d}\epsilon_t^{a*}}{\mathrm{d}\gamma} = -\frac{V_{\epsilon_t^a\gamma}}{V_{\epsilon_t^a\epsilon_t^a}} = -\frac{F'[\psi\tau(\beta+\kappa-\epsilon_t^{a*})y_t]\tau y_t[X-\xi(E_t^a D_t)^2]}{-(A+B+C+D)} > 0. \tag{D.2}$$

$$\frac{\mathrm{d}\epsilon_t^{a*}}{\mathrm{d}\psi} = -\frac{V_{\epsilon_t^a\psi}}{V_{\epsilon_t^a\epsilon_t^a}} = -\frac{V_{\epsilon_t^a\psi}}{-(A+B+C+D)} \gtreqless 0$$

$$\begin{aligned}
\text{with} \quad V_{\epsilon_t^a\psi} &= \tau(\beta+\kappa-\epsilon_t^{a*})y_t F''[\psi\tau(\beta+\kappa-\epsilon_t^{a*})y_t](\gamma+\psi)\tau y_t[X-\xi(E_t^a D_t)^2]\\
&+F'[\psi\tau(\beta+\kappa-\epsilon_t^{a*})y_t]\tau y_t[X-\xi(E_t^a D_t)^2]\\
&\tau(\beta+\kappa-\epsilon_t^{a*})y_t F'[\psi\tau(\beta+\kappa-\epsilon_t^{a*})y_t]\tau y_t 2\xi(E_t^a D_t) \gtreqless 0;
\end{aligned}$$

A sufficient condition for $\frac{\mathrm{d}\epsilon_t^{a*}}{\mathrm{d}\psi} < 0 \Leftrightarrow V_{\epsilon_t^a\psi} < 0$ is:

$$\frac{F'[\bullet]}{F''[\bullet]} < (\epsilon_t^{a*}-\beta-\kappa)(\psi+\gamma)\tau y_t. \tag{D.3}$$

As β or κ increase, $(\epsilon_t^{a*}-\beta-\kappa)$ decreases according to Proposition 2. This implies that the condition is less likely to be fulfilled when suspicions go up, at least if the equilibrium values of $F'[\bullet]$ and $F''[\bullet]$ do not change too much. That is the case when the realisations of government competence are more dispersed, i.e. the density function in Figure 1 is flatter (low elasticity of the competence), which is more likely to apply, for instance, to developing countries or new democracies. In short, the condition is more likely to be violated in countries with high levels of suspicion and flat competence density function. Then the intuitive result of more unsophisticated voters producing more manipulation holds.

Proposition 4:

$$\frac{\mathrm{d}\epsilon_t^{a*}}{\mathrm{d}E_t^a[\epsilon_t]} = \frac{d\epsilon_t^{a*}}{d\lambda}$$

$$= -\frac{V_{\epsilon_t^a E_t^a[\epsilon_t]}}{V_{\epsilon_t^a\epsilon_t^a}} = -\frac{B+D}{-(A+B+C+D)} < 1 \quad \text{and positive,} \tag{D.4}$$

E Straightforward results referred to at the end of the general solution in the model section

1. Government Cost Effect: Higher repayment costs r_t and higher reputation costs ξ reduce the optimal growth forecast by the state government at the equilibrium:

$$(i)\quad \frac{d\epsilon_t^{a*}}{dr_t} = -\frac{V_{\epsilon_t^a r_t}}{V_{\epsilon_t^a \epsilon_t^a}} < 0; \qquad (ii)\quad \frac{d\epsilon_t^{a*}}{d\xi} = -\frac{V_{\epsilon_t^a \xi}}{V_{\epsilon_t^a \epsilon_t^a}} < 0.$$

2. Government Benefit Effect: A higher ego rent X increases the optimal growth forecast by the state government at the equilibrium:

$$(iii)\quad \frac{d\epsilon_t^{a*}}{dX} = -\frac{V_{\epsilon_t^a X}}{V_{\epsilon_t^a \epsilon_t^a}} > 0.$$

3. Leverage Effect: A higher tax rate τ and a higher national income y_t decrease the optimal growth forecast by the state government at the equilibrium:

$$(iv)\quad \frac{d\epsilon_t^{a*}}{d\tau} = -\frac{V_{\epsilon_t^a \tau}}{V_{\epsilon_t^a \epsilon_t^a}} < 0; \qquad (v)\quad \frac{d\epsilon_t^{a*}}{dy_t} = -\frac{V_{\epsilon_t^a y_t}}{V_{\epsilon_t^a \epsilon_t^a}} < 0.$$

As for 1, if the cost of manipulating the government's state-specific growth forecast increases, the government will be more careful in expanding fiscal latitude in order to gain an electoral advantage. The effect of increasing the social costs of deficits was already captured in the different setting of the Shi and Svensson model, though not explicitly. An analogous result is obtained with respect to an increase in reputation costs. As for 2, the incumbent is willing to increase the manipulation, if there is a larger benefit from being re-elected. This implies that the government accepts additional costs of producing a deficit caused by the expansion of fiscal latitude, which, in turn, is the result of announcing a more optimistic government growth forecast. Despite the model differences, such an effect of ego rents on manipulations is also confirmed by Shi and Svensson. In 3, as τ or y_t increase, the effect of a government's growth forecast manipulation on fiscal latitude as well as on the deficit becomes larger. So the government should reduce its state-specific growth forecast to achieve the same optimal level of fiscal latitude. (The marginal effects with respect to τ and y_t are the same.) This leverage effect is not captured in Shi and Svensson or Lohmann because the increase in fiscal latitude is obtained in their model by manipulating the deficit directly. Here, the deficit is obtained residually.
